# Measurement of crystal growth velocity in a melt-quenched phase-change material

**DOI:** 10.1038/ncomms3371

**Published:** 2013-08-29

**Authors:** Martin Salinga, Egidio Carria, Andreas Kaldenbach, Manuel Bornhöfft, Julia Benke, Joachim Mayer, Matthias Wuttig

**Affiliations:** 1 I. Physikalisches Institut (IA) and JARA-FIT, RWTH Aachen University, Sommerfeldstraße 14, 52074 Aachen, Germany; 2 Gemeinschaftslabor für Elektronenmikroskopie, RWTH Aachen University, Ahornstraße 55, 52074 Aachen, Germany

## Abstract

Phase-change materials are the basis for next-generation memory devices and reconfigurable electronics, but fundamental understanding of the unconventional kinetics of their phase transitions has been hindered by challenges in the experimental quantification. Here we obtain deeper understanding based on the temperature dependence of the crystal growth velocity of the phase-change material AgInSbTe, as derived from laser-based time-resolved reflectivity measurements. We observe a strict Arrhenius behaviour for the growth velocity over eight orders of magnitude (from ~10 nm s^−1^ to ~1 m s^−1^). This can be attributed to the formation of a glass at elevated temperatures because of rapid quenching of the melt. Further, the temperature dependence of the viscosity is derived, which reveals that the supercooled liquid phase must have an extremely high fragility (>100). Finally, the new experimental evidence leads to an interpretation, which comprehensively explains existing data from various different experiments reported in literature.

## Abstract (web summary)

Fundamental understanding of the kinetics of phase transitions in phase-change materials has been hindered by challenges in the experimental quantification. Via an *in situ* laser reflectivity technique, Salinga *et al.* measure the crystal growth kinetics, revealing an extremely high fragility in the supercooled liquid.

## Graphical abstract

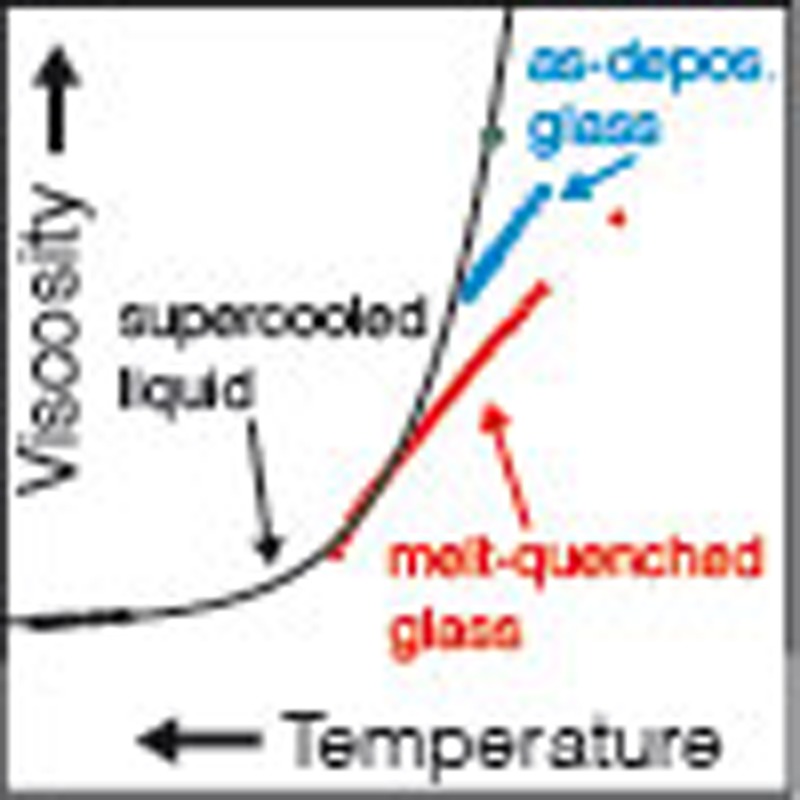

Crystallization phenomena are under investigation in several branches of material science, geology, chemistry and biology^1^. Although, for instance, the study and production of silicon single crystals has been the basis of today’s semiconductor industry, mineral formation continues to be one of the most intriguing topics within earth sciences. Among this wide variety of systems, the family of so-called phase-change materials represents a fascinating case, as crystallization can be observed here on a very small length scale of only a few nanometres and on an extremely short, that is, nanoseconds timescale^2^,^3^. Thanks to these unique switching properties, phase-change materials are already employed in high-density and ultrafast memories^4^. The data retention capability of those memories is determined by the stability of a melt-quenched amorphous volume against crystallization at low temperatures (typically up to 85 °C), whereas the maximum writing speed can be determined by studying the fast regime.

Experimentally, the measurement of crystallization kinetics on such short time and length scales is extremely demanding. Yet, such measurements are pivotal to predict the application potential of phase-change materials with respect to ultrafast data storage. Various groups have realized the potential of this topic and started investigations of crystal nucleation and growth employing *ab initio* molecular dynamics simulations. This is especially attractive because calculations with modern super computers already come very close to the volumes and timescales in which these phase transitions take place^5^,^6^.

Impressive progress has also been reported regarding the decrease of cell size, proving that phase-change memories of just a few nanometres in dimension can be switched between the amorphous and the crystalline state. On these length scales, nucleation becomes less important and crystallization will be governed by crystal growth. Hence, it is crucial to study crystal growth over a wide range of temperatures to forecast the stability of the amorphous state at low and intermediate temperatures, important to characterize retention, as well as high temperatures to predict the maximum switching speed. This is the goal of the present manuscript.

So far, measurements of crystal growth velocity have been limited to rather low temperatures where crystallization speeds are still slow^7^,^8^,^9^,^10^. It was only very recently that Orava *et al.*^11^ undertook a vast extension of the range of investigated crystallization speeds using ultrafast differential scanning calorimetry and derived information on the crystal growth velocity. In this study, the as-deposited amorphous phase was investigated under non-isothermal conditions. In the past, fast measurements have always been performed in a non-isothermal way employing short laser or voltage pulses to crystallize a small volume of material causing severe difficulties to obtain the temperature dependence of nucleation and growth velocities^12^,^13^,^14^,^15^.

In phase-change materials, crystallization can be so rapid that forming a disordered solid state requires very high quenching rates (10^9^–10^11^ K s^−1^) to avoid crystallization during the cooling of the liquid. As phase-change materials themselves typically show a comparably low thermal conductivity^16^, fast quenching is only possible by effectively draining off the heat into neighbouring materials. The larger the surface-to-volume ratio of the phase-change region, the better. In this way small volumes of a melt-quenched amorphous phase can be obtained. However, in such small volumes, it is quite difficult to perform precise quantitative experiments on crystallization kinetics. One reason is that many techniques need a minimum amount of material to produce enough detectable signal. Another issue is the inherent problem of achieving isothermal conditions during an experiment with such samples. Hence, the results obtained are specific for the sample geometry used, hampering the derivation of fundamental material properties^17^.

In this work, we take a new approach to quantify the temperature dependence of the crystal growth velocity *u*(*T*). Our laser-based method allows the investigation of the technologically relevant melt-quenched amorphous phase under isothermal conditions. At the same time, the new technique is capable of providing experimental data over a large range of crystal growth velocities (eight orders of magnitude) reaching up to the fastest regime (>1 m s^−1^). This unprecedented quantitative experimental evidence will lead to far-reaching conclusions about the viscosity of the material under investigation, here AgInSbTe, not only in the melt-quenched amorphous solid but also in its supercooled liquid phase.

## Results

### Laser reflectivity measurements

**Figure 1:**
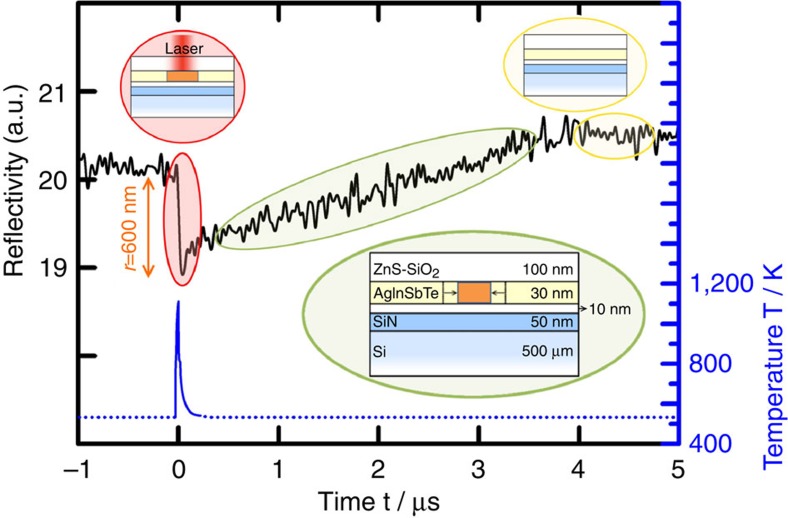
Time-resolved reflectivity measurement and simulated temperature profile. The black line is the reflectivity trace collected during a recrystallization experiment performed at a substrate temperature of 533 K. The zero of the timescale corresponds to the creation of the amorphous mark by the application of a laser pulse (83 mW for 30 ns at 658 nm wavelength). At this time, the reflectivity suddenly decreases (red ellipse) and then, because of the recrystallization process (green ellipse), it increases again up to a steady-state value (yellow ellipse) that corresponds to the complete recrystallization. The blue line shows the temperature profile during the laser irradiation process, simulated employing a finite element method (see Methods and Supplementary Note 4).

To determine the crystal growth velocity in a thin film of phase-change material, time-resolved reflectivity measurements with a bichromatic laser set-up were employed. An intense laser pulse is used to locally melt a cylindrical volume with a radius of several hundred nanometres in a thin crystalline phase-change film. In this study, AgInSbTe is chosen as material under investigation because it is successfully applied in data storage technologies and many relevant physical properties have been experimentally quantified for this alloy in the past. This material is sandwiched between two layers of transparent ZnS-SiO_2_ as depicted in the insets of Fig. 1. Once the laser pulse ends, the heat is very efficiently dissipated out of the melt into the Silicon substrate creating a melt-quenched mark. Finite element simulations of the layer stack show that it takes no longer than 100 ns to cool the phase-change material down to the temperature of the substrate (blue curve in Fig. 1). A second, low-intensity continuous-wave laser probes the reflectivity of the layer stack at exactly the same position where the first laser melts the phase-change material (black curve in Fig. 1). The reduction of reflectivity is a measure for how much of the previously crystalline material is amorphized. After thermalization, the gradual recovery back to a high reflectivity is thus an indicator for the progress of recrystallization. During the whole experiment, the sample is heated homogeneously at the temperature for which crystallization will be studied. The laser heating, however, is only used for the initialization of a crystallization experiment by creating an amorphous mark. This is crucial to obtain quantitative results for the temperature dependence of the crystal growth velocity using minimal assumptions. As the sample first needs to return to the substrate temperature, there exists an upper limit for the measurable crystallization speed. For AgInSbTe, this limit is reached at a temperature of ~550 K. To obtain meaningful data, the crystallization time must be significantly longer than the quenching time, that is, longer than 100 ns. This time corresponds to a crystal growth velocity of some m s^−1^. Although the collected data reach down to around 10 nm s^−1^, the present method can be even used for slower crystal growth velocities at the expense of longer measurement times. Hence, we can follow crystal growth over a wide range of temperatures.

### Confirmation of growth-dominated crystallization via TEM

**Figure 2:**
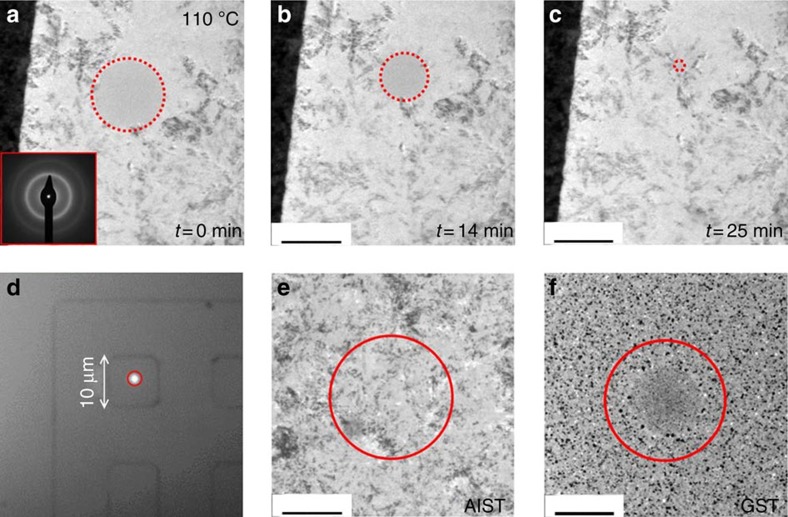
Recrystallization experiments probed by TEM. The first row shows a recrystallization experiment on AgInSbTe probed by *in situ* TEM during annealing at 383 K. From electron diffraction measurements on a selected area within the mark (inside the red dotted circle), one can infer that the material is completely amorphous there (inset of section **a**). The phase transition occurs via crystal growth from the amorphous–crystal interface. The radius of the amorphous bit decreases in time, up to the complete erasure. The figure sections **a**, **b** and **c** are also contained as frames in Supplementary Movie 1. That sequence of TEM images taken *in situ* at 383 K shows most convincingly that the recrystallization takes place by continuous growth from the rim. Section **d** has been obtained with the camera installed in our optical tester and it shows the gold frame that is used to find the recrystallized bit after the recrystallization experiments were performed in the fast regime. The white dot delimited by the red circle inside the square is the area illuminated by the probe laser. The same area has been partially melted by the pump laser and isothermally recrystallized at the substrate temperature. The TEM image of section **e** refers to the annealing of an AgInSbTe sample at 473 K. No structural contrast between the recrystallized bit and the crystalline matrix is visible. When the same experiment (493 K) is performed using Ge_2_Sb_2_Te_5_, the recrystallization is nucleation-dominated and the erased bit has a pronounced contrast in comparison with the crystalline matrix (**f**).

The second major source of complication in interpreting experimental data on crystallization kinetics, besides the lack of isothermal conditions, is the entanglement of nucleation and growth. This problem is overcome here as the recrystallization of the amorphized mark takes place solely by the growth of the crystal-to-amorphous interface from the rim to the centre. This has been verified by transmission electron microscopy (TEM). The top row of Fig. 2 reports a series of micrographs showing that recrystallization of a mark annealed *in situ* at ~383 K occurs exclusively by crystal growth from the rim. This kind of *in situ* analysis can obviously only be performed in the slow crystallization regime. To obtain experimental evidence for the growth domination also in the ultrafast regime, that is, at higher temperatures, *ex situ* TEM measurements were performed. An amorphized mark that is erased by growth of the surrounding crystallites should be indistinguishable from its crystalline environment. Figure 2e shows a TEM micrograph of an amorphous mark recrystallized at 473 K, a temperature in the middle of the investigated range. The contrast that is visible in this TEM image is solely because of a bending of the lattice inside large crystallites^18^,^19^. To verify this interpretation of the complex contrast variation visible in the bright-field TEM images, tilt series were acquired in the area comprising a bit recrystallized at 553 K (see Supplementary Note 5, Supplementary Fig. S7 and Supplementary Movie 2). A detailed analysis of these tilt series confirms that no structural difference is visible between the recrystallized bit and its environment. This feature, observed also in Fig. 2c, verifies that a growth-dominated process is also active in the fast regime at elevated temperatures. A very different TEM micrograph is expected if nucleation also has a considerable role in the crystallization process. This is confirmed by a comparable experiment performed for Ge_2_Sb_2_Te_5_, another phase-change material well-known from applications. Here nucleation interferes in the recrystallization process and the size of clearly distinguishable crystallites inside the bit is even smaller than that in the surrounding polycrystalline phase (see Fig. 2f).

Moreover, in our measurements on AgInSbTe, no lag time before the rise of reflectivity has been observed but recrystallization always started immediately after melt quenching. In contrast, comparable laser experiments in the ultrafast temperature regime show a minimum incubation time of at least 20 μs before nucleation^20^,^21^. From this difference, we can conclude that in our experiments the recrystallization process is dominated by crystal growth, even in the ultrafast regime.

### Determination of crystal growth velocity

The erasure of the amorphous bit in the film of phase-change material takes place by growth from the rim as verified in the above section. Thus, the crystal growth velocity at a given temperature can be calculated by dividing the radius *r* of the created mark at the beginning of the recrystallization process by the time it takes for the reflectivity to fully recover. The initial radius *r* is computed from the drop in reflectivity induced by the laser pulse while taking into account the intensity profile of the probe laser and the dielectric function of all materials in the layer stack. This method was cross-checked by comparing the radius resulting from this calculation with both *ex situ* transmission electron microscopic (TEM) analyses and finite element calculations of the laser-induced melting of the film (see Methods and Supplementary Note 4). A good quantitative agreement was found between the different techniques. In the light of the above explanations, it is apparent how the design of our experiment enables a data analysis making as few assumptions as possible. Experimental evidence has been presented that justifies each step of the analysis^17^,^22^.

### Crystal growth velocity

**Figure 3:**
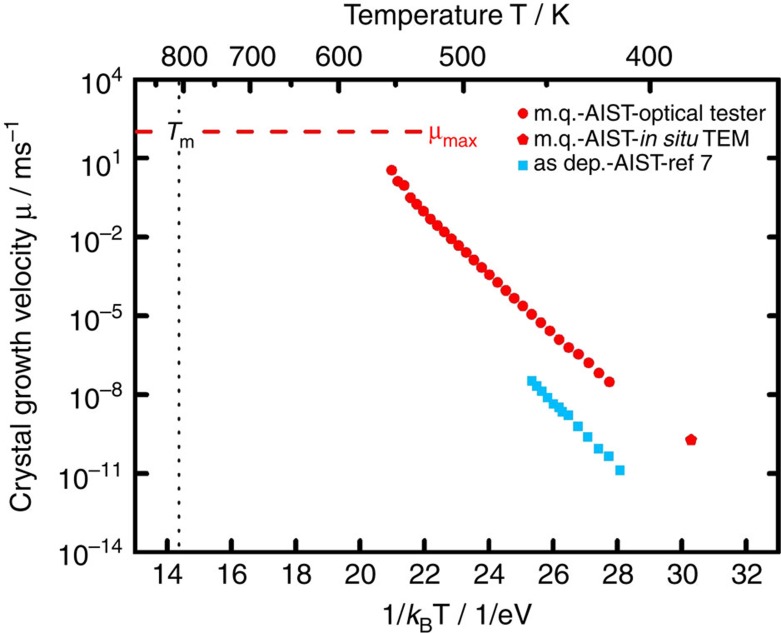
Temperature dependence of crystal growth rate. The growth velocity in melt-quenched amorphous AgInSbTe (red circles) has been measured by the optical tester on a wide range of temperatures probing more than eight orders of magnitude both in the slow and in the ultrafast crystallization regime. The red pentagon refers to the *in situ* TEM recrystallization experiment reported in Fig. 2. The data exhibit an Arrhenius dependence on temperature characterized by a unique activation energy of 2.7 eV. A similar behaviour has been measured over a much smaller range of velocities and temperatures in as-deposited blanket AgInSbTe thin films^7^ (blue squares). The maximum speed for melt-quenched AgInSbTe (~100 m s^−1^) has been estimated on the basis of the two-pulse experiments described in the Supplementary Note 1.

For AgInSbTe, our laser experiments result in the crystal growth velocity as a function of temperature between 418 and 553 K, as plotted in Fig. 3. The measured recrystallization times range from several seconds down to some hundred nanoseconds and correspond to crystal growth velocities spanning eight orders of magnitude from around 100 nm s^−1^ at 418 K up to >3 m s^−1^ at 553 K. At each temperature, the measurement was repeated 10–100 times to obtain good statistics. The fastest crystallizations might have been measured at temperatures a little higher than the substrate temperature, because the cooling after the melt-quench pulse was not complete, when the recrystallization took place. Still the experimental data fit nicely to an Arrhenius law (with activation energy of 2.7 eV), as one would expect for a glass. In the same figure, the data available in literature for AgInSbTe (ref. 7) are displayed. It is noteworthy that those measurements are performed in the as-deposited amorphous phase. Those data are not only restricted to the slow end of the growth velocity scale but they also extend over a much narrower range of temperature. The comparison with our data on melt-quenched samples shows that as-deposited films exhibit a much slower growth velocity at a given temperature. This demonstrates how crucial it is to measure *u*(*T*) for the technologically relevant melt-quenched state to derive realistic estimates of device performance. Indeed, different retention times and switching speeds for amorphous as-deposited and melt-quenched devices have already been reported for different phase-change materials, for example, Ge_2_Sb_2_Te_5_ and doped SbTe^23^,^24^,^25^. The data presented in Fig. 3 now provide an explanation for these observations.

Although the data in Fig. 3 end at 553 K for experimental reasons, it can be clearly seen that it is not possible to extend the Arrhenius behaviour of *u*(*T*) up to the melting point (*T*_m_=808 *K*). Assuming this trend, the speed of sound in amorphous AgInSbTe (~1,000 m s^−1^) would be overcome at around 625 K (see Supplementary Note 1). Further, using a two-pulse experiment, the maximum crystal growth velocity can be estimated to be between 10 and 100 m s^−1^ (for details see Supplementary Note 1). As previously stated, at 553 K a value of 3.4 m s^−1^ is measured, not very far from this maximum. Hence, at higher temperatures, a quite dramatic change in the temperature dependence of the crystal growth velocity must occur. It is reasonable to suspect that the origin of this change is related to the intrinsic characteristics of the disordered phase.

### Transfer to viscosity

The rest of this article will be devoted to the development of a comprehensive interpretation of the various observations on crystallization kinetics in phase-change materials. To this aim, we will discuss the glass dynamics of these alloys. Therefore, we derive values for the viscosity *η* of AgInSbTe from the measured crystal growth velocities *u*, employing the fact that the temperature dependence of *u* is tightly connected with the temperature dependence of *η*. To calculate the viscosity, the reversed form of the following equation is used:


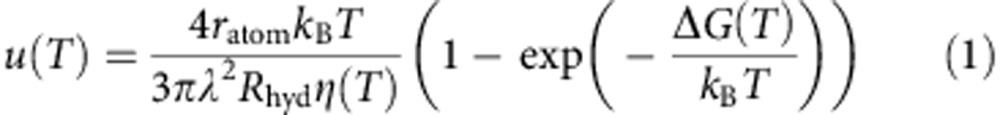


**Figure 4:**
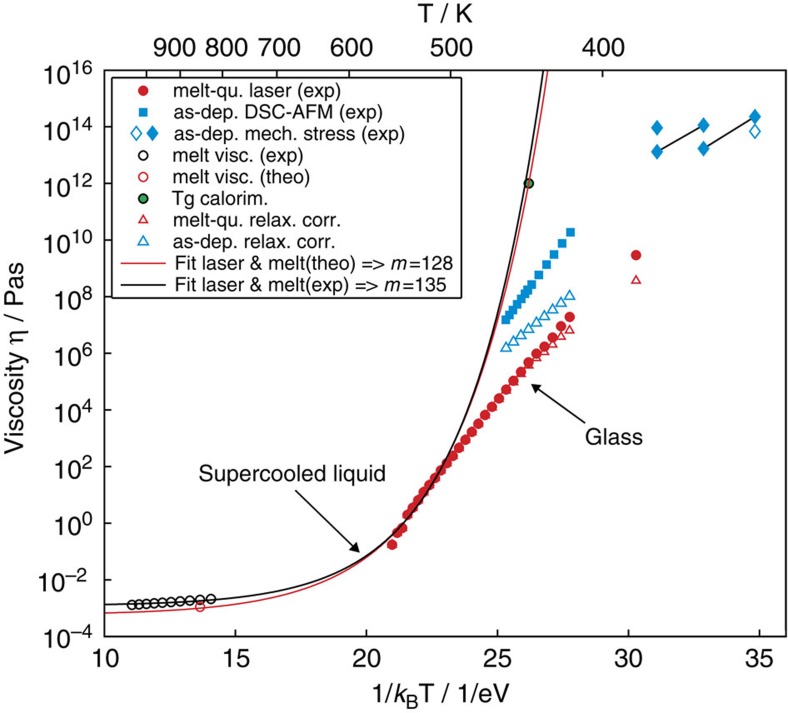
Temperature dependence of viscosity. Reversing equation (1) and using the growth velocity measurements of Fig. 3, we have calculated the viscosity of AgInSbTe as a function of temperature (filled red circles). The general understanding that a glass is formed upon cooling from a supercooled liquid implies that the curves of supercooled liquid (continuous lines) and glass (red triangles) have to connect. The lines (black and red) are obtained by fitting the equation proposed by Mauro *et al.*^38^ for the description of the viscosity of a supercooled liquid to the laser results at the 11 highest measured temperatures, together with literature values for the viscosity in the liquid phase (in case of the black line the experimentally determined viscosities in liquid Sb_80_Te_20_, an alloy close to the base stoichiometry of the AgIn-doped SbTe used in the present work, from ref. 26 represented by the open black circles and in case of the red line the theoretically derived viscosity of AgInSbTe from ref. 27 represented by the red open circle). Both fits correspond well with a viscosity of 10^12^ Pas at the glass transition temperature *T*_g_=443 K (green-filled circle) that was previously observed for AgInSbTe using calorimetry^39^. The blue squares are obtained using the data on as-deposited AgInSbTe reported in ref. 7. The blue diamonds are extracted from ref. 32 in which viscosity of AgInSbTe has been measured via stress relaxation. The filled blue diamonds represent the viscosity of annealed amorphous samples, the open diamond states the original value before annealing. The original viscosity values derived from growth velocities (red filled circles from current laser experiments and blue filled squares from previous studies on as-deposited amorphous AgInSbTe) are corrected for structural relaxation they had time for during the experiments resulting in open red and open blue triangles, respectively.

Here *r*_atom_ is the atomic radius (the half of the bond length, 1.5 Å), *λ* is the diffusional jump distance (~1 Å), *η*(*T*) is the viscosity of the disordered surrounding, *k*_B_ is the Boltzmann constant, Δ*G*(*T*) is the Gibbs energy difference between the liquid and the crystalline phase, *R*_hyd_ is the hydrodynamic radius (~0.5 Å). The latter has been estimated by the Stokes–Einstein equation, applied at the melting point, using the data of viscosity and atomic diffusivity reported in refs 26, 27. The Gibbs energy gain has been estimated using the Thompson–Spaepen approximation^28^


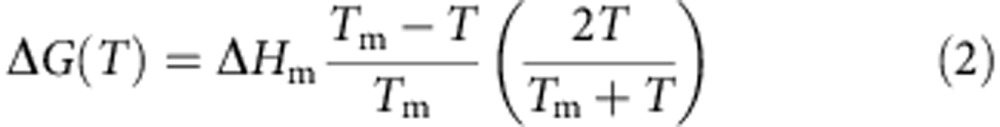


for which the heat of fusion Δ*H*_m_ (173±3.1 meV/at) and the melting temperature *T*_m_ (808 K) have been extracted from ref. 29. In the temperature range probed, the change of the exponential term in equation (1) is so small (that is, 0.9<Δ*G*(*T*)/*k*_B_*T* <1.6) that it cannot account for more than a change of 26% in *u*(*T*). This clearly shows that the change of *u*(*T*) over eight orders of magnitude is dominated by the temperature dependence of *η*(*T*). Hence, the viscosity also exhibits an Arrhenius dependence on temperature, as can be seen in Fig. 4. Here the results of the calculation are displayed as red dots (blue squares for as-deposited data from ref. 7). For this calculation, all parameters in equation (1) have been chosen to ensure that the viscosity is not underestimated. As for *u*(*T*), also for *η*(*T*), the experimentally determined Arrhenius behaviour cannot extend to much higher temperatures, as the viscosity value at around 550 K (~170 mPas) is not even two orders of magnitude away from the viscosity measured in the liquid (~2 mPas)^26^. Such a pronounced flattening out of *η*(*T*) (and thereby also of *u*(*T*)) towards higher temperatures can only be realized if a material’s supercooled liquid phase has a high fragility. This will become most evident in the progress of this article. The strong bending of the *η*(*T*)-curve that comes with a high fragility is, however, incompatible with the straightness of the experimental data at lower temperatures. This is true even if one takes into account a potential decoupling of the viscosity from the atomic diffusivity *D*. The latter is the physical quantity that actually controls crystallization processes. *D* is commonly related to the viscosity *η* by the Stokes–Einstein equation *D*(*T*)
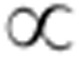
*k*_B_*T*/*η*(*T*), which had already implicitly been applied in equation (1). Therefore, when in other fragile liquids the temperature dependence of crystallization rates towards lower temperatures were observed to straighten out more than the viscosity of the supercooled liquid, this was attributed to a decoupling of atomic diffusivity from viscosity^11^,^30^. It has been argued that when a fragile liquid is cooled towards *T*_g_, local relaxation occurs at substantially different rates at different places, generating nanometric areas with varying atomic diffusivities *D* (ref. 31). According to this model, the Stokes–Einstein equation breaks below ~1.2 *T*_g_, and it should be modified by assigning an exponent *ξ*≤1 to *η*: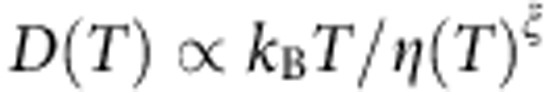. However, for our data, such an adaptation of equation (1) is not able to explain the measured crystal growth velocities in AgInSbTe, especially not the strict Arrhenius-like temperature dependence (see Supplementary Note 2 and Supplementary Fig. S2). As our experimental data cannot be described by the theoretical formulas for a supercooled liquid phase, we consequentially interpret them as representing a melt-quenched glass for which the atomic configuration deviates from the equilibrium configuration of the supercooled liquid at a rather high temperature owing to the vast cooling rates employed.

**Figure 5:**
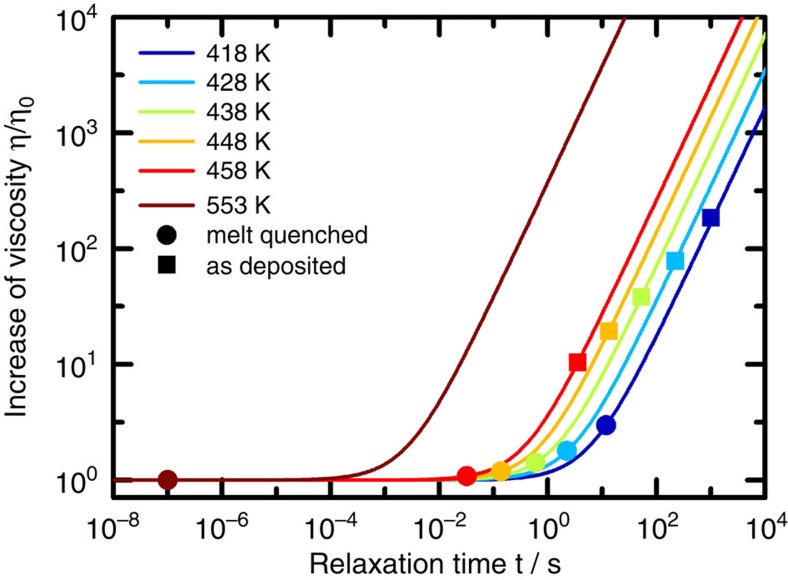
Temporal increase of viscosity due to structural relaxation. The lines show the time evolution of viscosity at different temperatures (different colours) according to equation (3), in which the parameters have been chosen to reproduce the data reported in ref. 32. The filled circles indicate half of the recrystallization time of melt-quenched amorphous bits, whereas the squares refer to the incubation time before nucleation in as-deposited blanket films^8^, both at the respective experimentally realised temperature (indicated by the colour).

As glasses are non-equilibrium solid states, next we need to consider the implications of structural relaxation for experimental determination of crystallization rates. Far from equilibrium, the temporal increase of viscosity in a glassy state of a phase-change material can be described as^32^,^33^


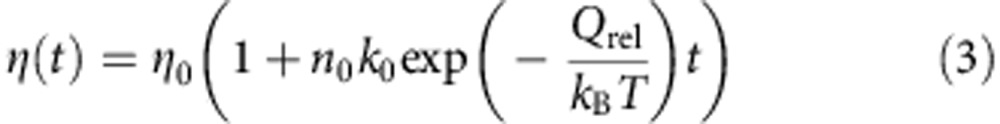


an equation derived for bimolecular relaxation dynamics, where *η*_0_ is the initial value of the viscosity. From an experimental determination of the viscosity’s temporal evolution^32^ in AgInSbTe, we take *Q*_rel_=1.14 eV and derive the product 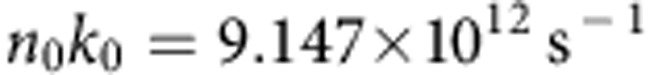 from 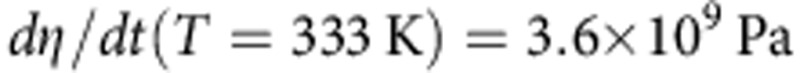 and 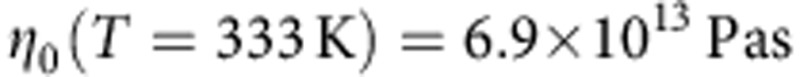. In a crystal growth experiment, such as the one in the present study or the one presented in ref. 7, depending on the period of time a sample was kept at an elevated temperature to measure crystal growth velocity, the amorphous phase could relax significantly towards higher viscosities during the experiment. This turns out to be especially important for measurements of crystal growth velocity in the amorphous as-deposited state. Here the samples are kept at elevated temperatures for a non-negligible incubation time to form nuclei, whose growth is then measured. However, a series of data points in an *η*(*T*) diagram (such as the blue squares or red circles in Fig. 4) is usually understood to describe a fixed amorphous configuration of material without structural relaxation just at different temperatures (iso-configurational line). Thus, it is necessary to correct the values of viscosity by as much as the viscosity had time to increase during a certain measurement. Figure 5 illustrates how viscosities need to be corrected according to equation (3) applying the experimentally determined parameters given above. Although equation (3) overestimates the increase of viscosity on relaxation close to equilibrium, that is, where an amorphous state is close to the supercooled liquid, at lower temperatures on the other hand the supercooled liquid phase will turn out to be far away from the investigated amorphous phase in all experiments^34^. Therefore, in the case of the experiments on as-deposited samples (squares), the viscosities need to be corrected by up to more than two orders of magnitude. This is different for the viscosity data of the melt-quenched state (dots), which only require noticeable correction at the lowest temperatures. The reason for the corrections being very small for the melt-quenched state lies in the concept of the new laser method, that is, in measuring recrystallization purely by growth from a pre-existing crystalline interface; hence, there is no waiting time between preparation of the amorphous state and the actual measurement. The corrected viscosities are added into Fig. 4 both for melt-quenched (red triangles) and as-deposited amorphous (blue triangles). For all temperatures, the corrected viscosity values now consistently represent the viscosity at the beginning of the first anneal at each temperature, and can therefore consistently be compared among different temperatures.

To further validate the transfer of experimental data on crystallization kinetics into the temperature dependence of viscosity, viscosity values from measurements of mechanical stress relaxation on as-deposited amorphous AgInSbTe are included into Fig. 4 (blue diamonds)^32^. It is affirming to recognize that the series of viscosity data derived from crystal growth in the as-deposited phase (blue triangles) points quite nicely across many orders of magnitude towards the value of viscosity in a hardly relaxed as-deposited sample at 333 K (open blue diamond). This agreement is especially remarkable, as the latter originates from a completely different kind of experiment, which is more directly linked to the physical material property viscosity. The slight inaccuracies in matching of these two types of experimental measurements might well result from a difference in age (storing duration at room temperature) the as-deposited samples had, when being investigated. After we have convinced ourselves of the quantitative correctness of our viscosity data, in the following we will proceed with considering their implications.

### Viscosity of the supercooled liquid

The temperature dependence of viscosity in the supercooled liquid phase can vary significantly between different materials. In so-called ‘strong’ glass-formers, an increase in temperature above the glass transition temperature *T*_g_ causes a very steady decrease in viscosity, following quite strictly an Arrhenius law. In other so-called ‘fragile’ materials, however, viscosity drops in a much more pronounced way right above the *T*_g_. The kinetic fragility *m* quantified as the steepness of *η*(*T*) at the glass transition temperature *T*_g_, that is, *m*=[∂log_10_(*η*)/∂(*T*/*T*_g_)]_*T*=*T*g_, can thus act as a measure for the ‘degree of deviation’ of *η*(*T*) from an Arrhenius behaviour^35^. Fragilities reported in literature range from 20 for very strong liquids like SiO_2_ up to over 150 for some very fragile, typically organic polymers (inorganic materials show fragilities up to m~90)^36^,^37^.

As can be nicely seen in Fig. 4, to bring the viscosity of the supercooled liquid phase of AgInSbTe up from the viscosity of the liquid phase^26^ (around 10^−3^ Pas) in a way that it is in line with the values derived from our measurements, extremely high fragilities are necessary. The general understanding that a glass is formed on cooling from a supercooled liquid implies that the curves of supercooled liquid (continuous lines) and glass (red triangles) have to connect. Using the equation proposed by Mauro *et al.*^38^ for the description of the viscosity of a supercooled liquid, the fragility turns out to be *m*
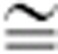
135 (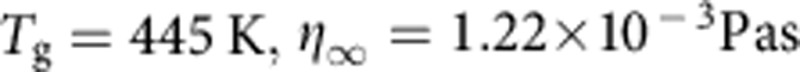) fitting the laser results at the highest temperatures together with the experimentally determined viscosities in liquid Sb_80_Te_20_ from ref. 26 (open black circles). Fitting the same laser results together with a value 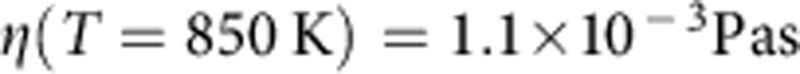 for the viscosity of liquid AgInSbTe as derived in theoretical simulations (open red circle, data taken from Supplementary Information for ref. 27) still results in a fragility of *m*
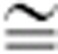
128 (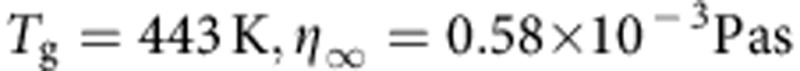). Both fits correspond well with a viscosity of 
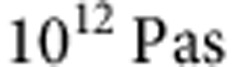
 at the glass transition temperature *T*_g_=443 K that was previously determined for AgInSbTe using calorimetry^39^. In case the more traditional Vogel–Fulcher–Tammann formula is preferred to describe *η*(*T*) in the supercooled liquid^40^, the fragility would even go up to *m*
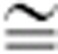
190 to be in accordance with the experimental data (see Supplementary Note 3).

Although our experimental data necessitate the supercooled liquid to be highly fragile, the theoretical formulas for supercooled liquids fail to describe the whole set of experimental data (even when decoupling of diffusivity and viscosity is considered, as mentioned above and illustrated in Supplementary Fig. S2). Therefore, unlike the supercooled liquid curve, the blue and red triangle symbols in Fig. 4 represent two fixed (but mutually different) configurations of the glass (iso-configurational line). In this light, the difference between viscosities (and crystal growth velocities) in the melt-quenched and the as-deposited state can be attributed simply to a different configuration or degree of relaxation. In that sense, an amorphous state that is produced by quenching from the melt in <100 ns can be expected to be a highly unrelaxed glass, apparently less relaxed than an amorphous sample that was produced by sputter deposition and crystallized at a considerably later time. In this way, the faster crystallization in the melt-quenched amorphous state can be understood.

It is well-known that when cooling down a liquid, a faster cooling rate causes an earlier glass transition, that is, at higher temperatures^41^. As a consequence, in the case of AgInSbTe, the data from the laser experiments turn out to lie quite tangential to the supercooled liquid curve in the higher temperature range of the measurement. Therefore, although the experimental data follow an Arrhenius law quite well, as one would expect for an amorphous solid, at higher temperatures it cannot unambiguously be distinguished whether the experiments took place in the supercooled liquid phase (theoretical curves) or in the amorphous solid (Arrhenius law). For a phase-change material like AgInSbTe, it seems extremely difficult to probe crystal growth velocities unambiguously in the supercooled liquid state, as slower quenching rates allow crystallization to interfere, whereas increasing the cooling rate will result in a glass transition at even higher temperatures. It is fascinating that the fragility of the supercooled liquid phase has such a great influence on the crystallization properties of a phase-change material, and at the same time, this fundamental physical property is not directly accessible.

## Discussion

The outcome of a very high fragility for AgInSbTe resulting from the above interpretation is in line with a generally observed correlation between a high fragility and a large difference in configurational entropy or heat capacity between the liquid and amorphous phase of a material^35^,^42^,^43^. It is remarkable though, that other materials with high fragility, i.e. typically organic/molecular compounds, generally show very slow crystallization kinetics because of their rather cumbersome building blocks that need to be rearranged, while the corresponding driving forces per unit (or per mol) are not so high (intermolecular interactions via van-der-Waals forces are rather weak). Therefore, it seems that it is the unusual combination of low viscosity in the liquid, small building blocks and significant driving forces (all not uncommon for inorganic materials), together with an extremely high fragility that opens up a wide temperature window of low viscosity and thus high crystallization speeds. How can a material have such a high fragility although it has no apparent indestructible and cumbersome building blocks? Our present work raises this fundamental question that may lead towards a deep understanding of the microscopic origin for the central property of phase-change materials: their phase-change characteristics. At the same time, the presented comprehensive description of the viscosity of the disordered phases of AgInSbTe is itself already meaningful for memory technologies based on phase-change materials.

Both switching speed and data retention of phase-change memories are directly related to crystallization kinetics. For the first time, direct experimental evidence for crystal growth velocities reaches so far up into the fast regime, providing solid grounds for improved simulations of the switching processes in memories. On the low-temperature side, our work makes apparent how much the stability against crystallization differs depending on whether it is determined in as-deposited or in melt-quenched amorphous phases. Our method is an ideal tool to investigate the technologically relevant phase. Further, a careful consideration of the influence of structural relaxation is shown to be of great importance also for crystallization kinetics of phase-change materials, not only for their electrical properties^33^,^44^,^45^. Studying the viscosity of the amorphous phases will thus certainly have an impact on the field of memory applications. However, this system, that is, the combination of material class and experimental scheme, seems to be also highly instructive for a fundamental understanding of supercooled liquids and glasses in general.

## Methods

### Laser reflectivity measurements

For locally melting the thin crystalline phase-change film, a 30-ns-long pulse from a laser with a wavelength of 658 nm is used arriving at the sample with a power of 83 mW. The second, low-intensity laser for probing the reflectivity has a wavelength of 639 nm. It continuously illuminates the sample with only 0.1 mW. The alignment of both lasers is achieved by combining both beams into one single-mode optical fibre before focusing it on the sample surface through a microscope objective. This also helps ‘cleaning’ the intensity profiles of both beams into Gaussian distributions. The sample temperature is controlled with a heated sample holder—similar to a hot plate—using a thermocouple to check the temperature during the experiment with an accuracy of ±1 K.

### Transmission electron microscopy

For all TEM measurements in this work, a FEI Tecnai F20 has been used (mainly) in bright-field TEM mode. To ensure that the TEM analysis is performed for the exact region exposed to the laser, a gold frame was lithographically fabricated on the sample surface before a laser experiment. The gold frame is large enough (10 μm) to not affect the laser measurement (see both gold frame and laser spot in Fig. 2d). Information about how an electron transparent film was prepared out of a sample after laser experiments have been performed are given in the methods section on sample preparation below.

### Simulations using finite element method

For simulating the thermal response of our sample to laser heating, we employed the finite element method ( http://www.comsol.com; accessed 25 July 2013). The heat conduction equation takes the form


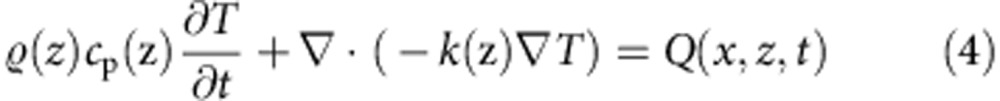


where *ρ* is the density, *c*_p_ the specific heat and *k* the thermal conductivity. The heat source 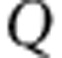 we describe as


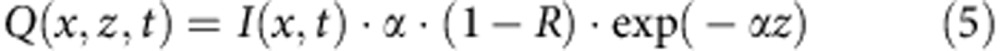


The spatial and temporal profile of the power density *I* of the pulse laser used has been measured with a charge-coupled device and a fast photodiode. A power-meter has been used to determine the total power released at the position of the sample. The reflectivity *R* has been directly measured by a spectrophotometer, whereas the absorption coefficient *α* has been calculated taking into account multiple reflections at the various interfaces of the multilayer stack on the basis of the refractive indices reported in refs 46, 47, 48. The specific heat, the density and the thermal conductivity of the different materials, as function of temperature, have been extracted from refs 43, 49, 50, 51. The melting process has been described taking into account the heat of fusion^52^. The thermal boundary resistance between AgInSbTe and ZnS-SiO2 has been included on the basis of the data reported in ref. 53 for GeSbTe.

### Sample preparation

A crucial aspect for the recrystallization experiments is to choose a suitable layer stack. A capping material should be applied to the layer stack to prevent evaporation and oxidation of the phase-change layer during the heating. In analogy to the layer stack of optical discs, (ZnS)_80_-(SiO2)_20_ has been selected to encapsulate the Ag_4_In_3_Sb_67_Te_26_ layer. This way, practically no laser light is absorbed in the capping layer. To be able to rapidly cool the locally molten phase-change material down for isothermal experiments at the temperature of the heated stage, it is absolutely necessary to have a large enough heat sink in close proximity to the phase-change material. Therefore, silicon with its very high thermal conductivity has been chosen as a substrate. In addition, sufficient optical contrast is required to be able to distinguish the reflectivity of an amorphous bit from the reflectivity of a fully crystalline film. This is achieved by optimizing the thicknesses of the different layers in the layer stack. A thin SiN layer has been deposited on top of the Si substrate to allow an easier preparation of the TEM samples. To obtain an electron transparent film, after the mechanical dimple grinding, the last few micrometres have been chemically removed. To this aim, KOH has been used as an etchant of the silicon substrate. The SiN layer, working as an etch stop, protects the phase-change layer. A sketch of the sample structure is reported in the inset of Fig. 1. The phase-change film has been direct current. sputter-deposited with an LS 320 von Ardenne system (background pressure 2 × 10^−6^ mbar, 20 s.c.c.m. Ar flow, deposition rates 0.1 nm s^−1^) operating in constant power mode (20 W) using stoichiometric targets of 99.99% purity.

## Author contributions

M.S. conceived the method and designed the study. A.K., J.B. and E.C. produced the samples and performed the laser experiments. TEM measurements were performed by M.B. and interpreted by M.B., J.M., E.C., M.S. and M.W. E.C. performed the computer simulations. A.K., E.C. and M.S. analysed the results on crystal growth velocities. M.S. and E.C. interpreted all results and wrote the manuscript. M.W. contributed to the discussion presented in the article and edited the manuscript.

## Additional information

**How to cite this article:** Salinga, M. *et al.* Measurement of crystal growth velocity in a melt-quenched phase-change material. *Nat. Commun.* 4:2371 doi: 10.1038/ncomms3371 (2013).

## Supplementary Material

Supplementary Information Supplementary Figures and Notes

Supplementary Movie 1 TEM bright field images showing the erasure of an amorphous bit in AgInSbTe during an in-situ annealing at 383K.

Supplementary Movie 2 TEM tilt series covering a tilt range of +/- 20 degree at an increment of 1
