# Supplementary material for: Measurement of crystal growth velocity in a melt-quenched phase-change material
Source: Nat Commun. 2013 Aug 29;4:2371. doi: 10.1038/ncomms3371 (PMC3763494; doi:10.1038/ncomms3371)
Supplement: Supplementary Information — Supplementary Figures and Notes [file ncomms3371-s1.pdf]

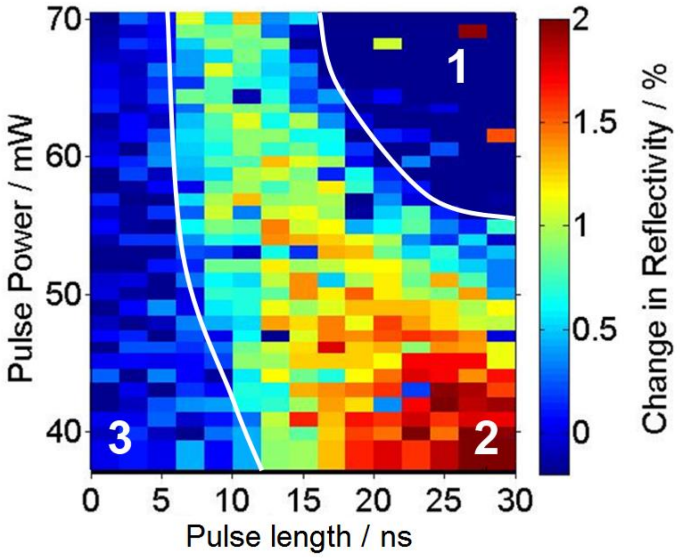

Supplementary Figure S1 | Power-time-effect for the recrystallization of melt-quenched amorphous bits (erase process with substrate at room temperature). Three different zones can be distinguished. In zone 1 the erase pulses lead to local melting. Sub-sequent quenching results in re-amorphization of the film. Partial recrystallization is obtained in area 2 whereas in area 3 the power is not enough to induce crystal growth in this timescale.

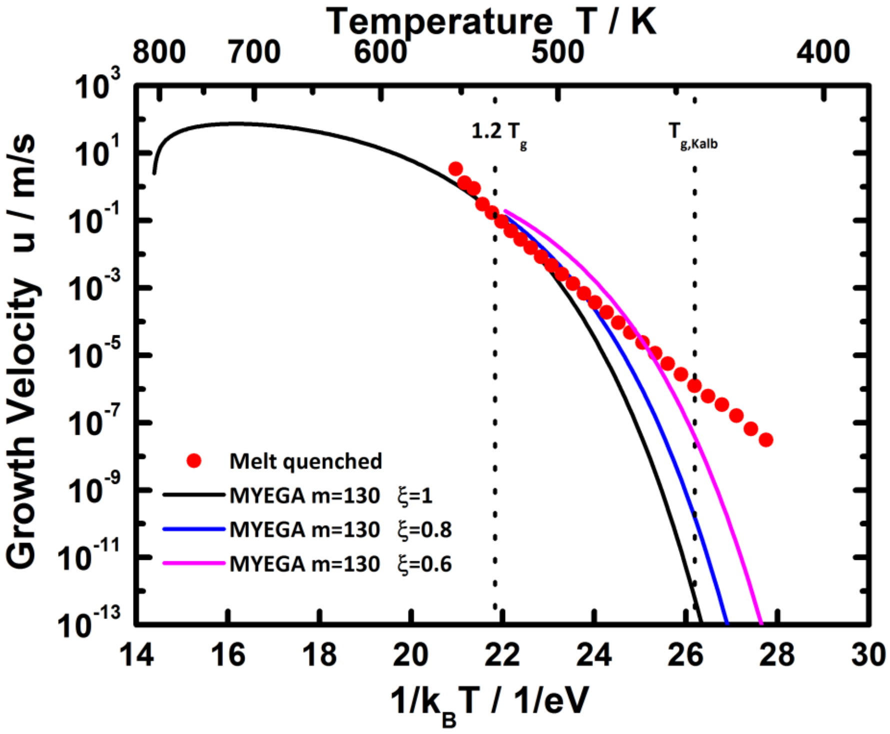

Supplementary Figure S2 | Temperature-dependent growth velocity and MYEGA model. The crystal growth velocity as calculated based on equation (1) and the MYEGA model for $\eta(T)$ (lines) does not match the experimental results (red dots) especially in the regime of slow crystallization at lower temperatures. Below 1.2 T_g an exponent $\xi = 0.6$ (magenta) or $\xi = 0.8$ (blue) has been assigned to the viscosity to take into account the decoupling effect according to Ediger et al. [30].

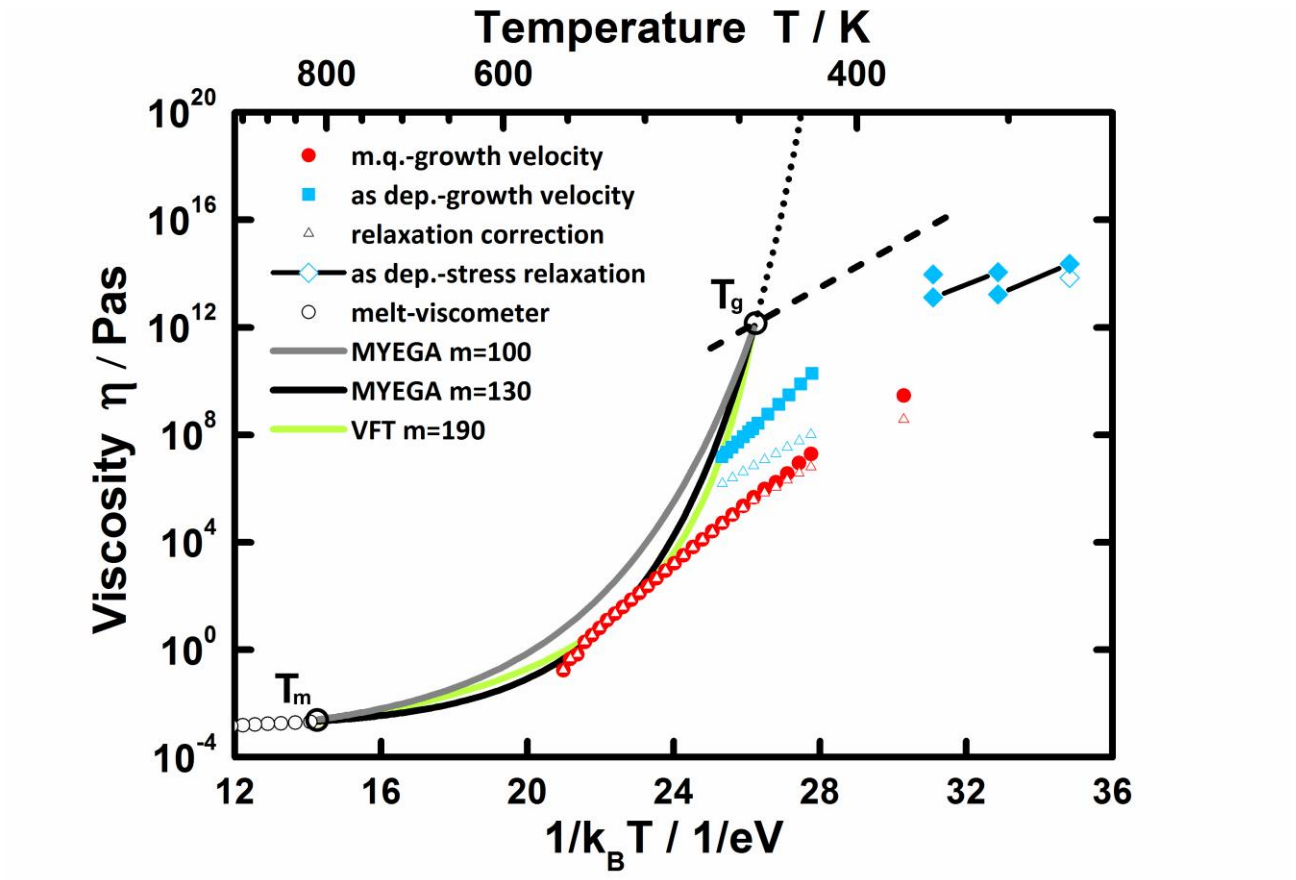

Supplementary Figure S3 | Temperature dependence of viscosity. Reversing equation (1) and using the growth velocity measurements of figure 3 we have calculated the viscosity of AgInSbTe as a function of temperature (red circles). The blue squares have been obtained using the data on as-deposited AgInSbTe reported in ref. [7]. The blue diamonds have been extracted from ref.[32] in which viscosity of AgInSbTe has been measured via stress relaxation. The black and grey lines have been obtained by the MYEGA equation[38] using a fragility of 130 and 100, respectively. The green line has been calculated by the VFT model assuming m=190. The glass transition temperature T_g has been fixed to 443K as measured by conventional DSC on an as-deposited and then relaxed AgInSbTe sample at a heating rate of 40 K/min[39]. The parameter η_∞ has been chosen to fit the viscosity of the liquid phase (black circles) reported in ref. [26] for $Sb_{80}Te_{20}$, an alloy close to the base stoichiometry of the AgIn-doped SbTe used in the present work.

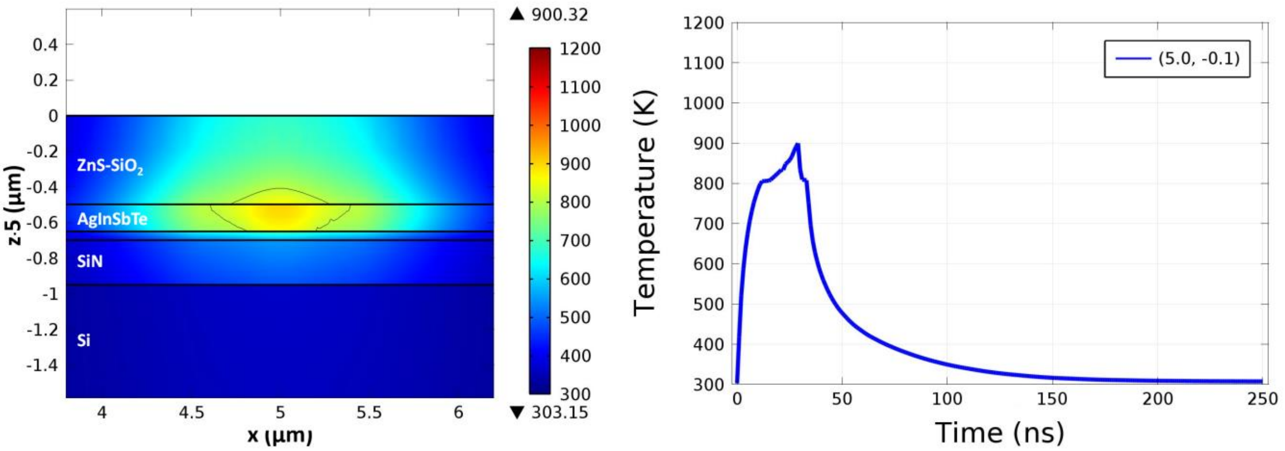

Supplementary Figure S4 | Calculated temperature profile for substrate temperature at 303K. The left panel shows a cross-sectional view of the temperature profile inside the AgInSbTe sample during the laser irradiation process. The image refers to the time t=29ns (i.e. just before the laser is switched off). The z axis has been enlarged by a factor of 5. The black line defines the area at $T>T_m$. The right panel shows the temperature at the centre of the irradiated region as function of time.

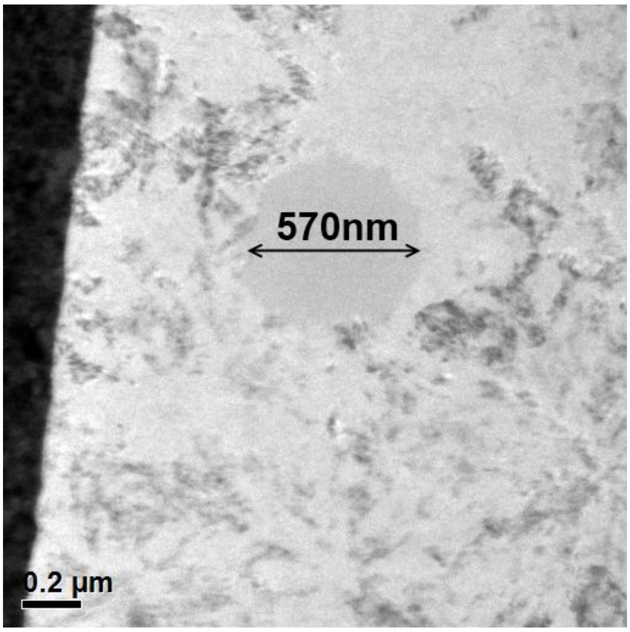

Supplementary Figure S5 | Transmission electron microscopy of an amorphous bit. The pump laser parameters have been set to reproduce the FEM calculation reported in fig. S4. The radius of the bit agrees well with the value expected from the simulation.

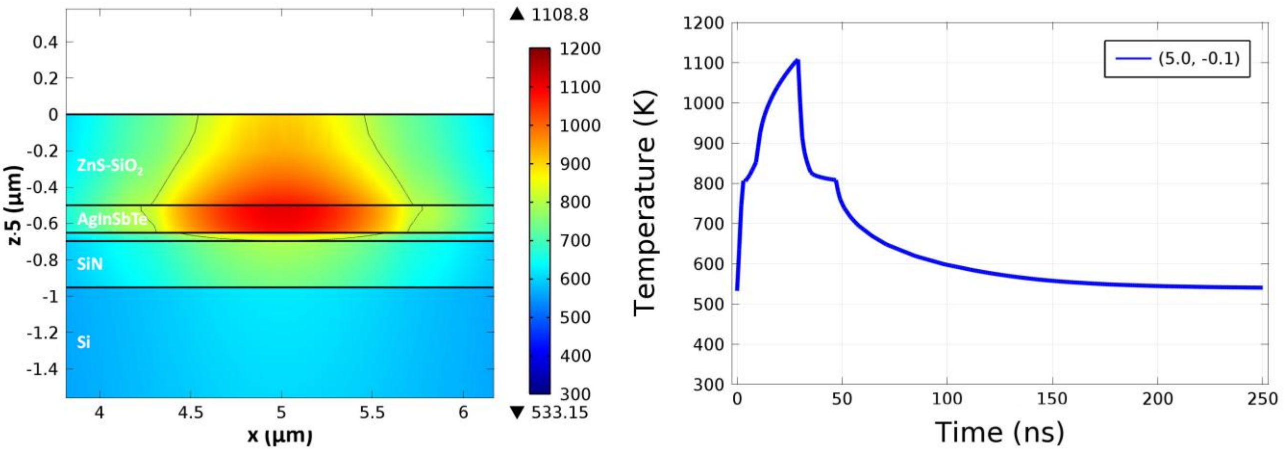

Supplementary Figure S6 | Calculated temperature profile for substrate temperature at 533K. The left panel is a cross-sectional view of the temperature at t=29ns. The right panel shows the temperature as function of time at the centre of the irradiated region. The size of the melted volume is in good agreement with the value expected looking to the drop in reflectivity reported in fig.1 of the main article.

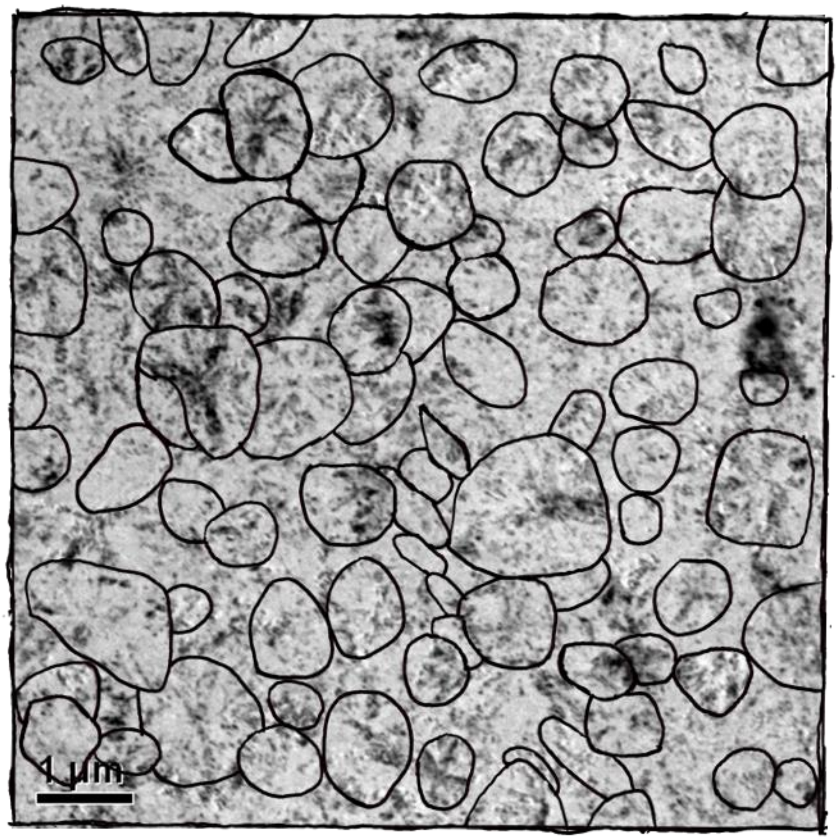

Supplementary Figure S7 | Identification of separate grains in AgInSbTe based on tilt series in TEM. The transmission electron microscopy bright-field image is part of a tilt series (supplementary movie 2) of an isothermally (at 533 K) recrystallized AgInSbTe sample at tilt angle 0 degree. The tilt series was performed in increments of 1 degree between -20 degree and +20 degree. The outlines of identified grains are drawn by hand in overlay. The grains were identified observing the movement of the bending contours during the tilt series. Small mismatch between the outlines of the grains and the TEM image occur due to sample drift during the tilt series.

SUPPLEMENTARY NOTE 1: Maximum crystal growth velocity

A two-pulses laser experiment has been performed in order to estimate the maximum crystal growth velocity of the material under investigation, AgInSbTe. At the beginning, the film is totally crystalline and the substrate is at room temperature. A first pulse is used to create an amorphous mark and the corresponding drop in reflectivity (-3.5%) can be used to calculate the radius of the bit as described above. A second pulse is used to recrystallize the sample and the increase in reflectivity provides information about the erasure process. Supplementary Figure S1 shows the result of this experiment in a power-time-effect (PTE) diagram. The PTE graph can be divided in three areas. In zone 1 the erase pulses lead to a local melting. Subsequent quenching results in a re-amorphization of the phase change material arriving at roughly the same reflectance the layer stack had after the first laser pulse. Erase pulses in zone 2 partially crystallize the amorphous bit created by the first laser pulse, so the reflectance after both pulses is still smaller than the original reflectance of the fully crystalline phase. A change in reflectivity of +1.5% corresponds to a movement of the amorphous-to-crystalline interface by ~100 nm during the laser annealing. For a pulse duration of 10ns this corresponds to a velocity of 10 m/s. In the part of zone 2 that borders zone 1, the annealing temperature is close to the melting point. The pulses in zone 3 cannot induce any significant recrystallization.

It must be noted that during the laser irradiation we are far from isothermal conditions and the temperature profile is not uniform inside the amorphous area. As a consequence, it is reasonable to assume that we are overestimating the annealing time when considering it to be equal to the pulse duration. On the other hand, a maximum crystal growth velocity much higher than 100m/s can be excluded. If that were the case a complete erasure of the amorphous bit should be possible in few nanoseconds. Therefore, we believe that the maximum crystal growth velocity in melt-quenched amorphous AgInSbTe should be between 10 and 100 m/s, which is not so far from the value measured at 553K (i.e. 3.4 m/s). In any case, we can be sure that the maximum speed in amorphous

AgInSbTe cannot overcome the speed of sound. It is known that the latter is related to the solid's density ρ, to its biaxial modulus Y and to the Poisson's ratio ν according to the equation

$$u_{sound} = \sqrt{\frac{Y}{(1-\nu)\rho}} \qquad \text{(S1).}$$

Using experimental data reported in literature ($\rho = 6.36\frac{g}{cm^3}$, $Y = 10$ GPa) [32] and assuming a Poisson's ratio equal to 0.3, the speed of sound for AgInSbTe approaches a value of ~1000 m/s.

To conclude, with respect to Fig. 3 of the main article it appears to be impossible for the Arrhenius behavior of u(T) to continue up to the melting point, since assuming this trend approaching 625 K the crystal growth velocity would increase beyond both the maximum speed obtained by the two-pulses experiments and the speed of sound in amorphous AgInSbTe. This means that, increasing temperature over 553K, the crystal growth velocity must saturate at temperatures significantly below the melting point, which can be explained by a high fragility in this alloy's (supercooled) liquid phase.

SUPPLEMENTARY NOTE 2: Is a decoupling between viscosity and atomic diffusivity in the supercooled liquid able to explain our data?

It has been demonstrated that several fragile liquids exhibit a decoupling between crystal growth kinetics and viscosity[30] most evidently approaching the glass transition temperature T_g. This phenomenon has been explained by Ediger et al. to be due to spatially heterogeneous dynamics[31]: When a liquid is cooled towards T_g, local relaxation occurs at substantially different rates at different places, generating nanometric areas with different atomic diffusivity. The decoupling between viscosity and diffusivity is seen as a proof of this heterogeneous dynamics. This behaviour is more evident in liquids with a high fragility. The data reported in literature about organic and inorganic liquids, however, refer to alloys in which crystallization is much slower than in the case of phase change materials[30]. This allows the measurement of viscosity, growth velocity and sometimes also the atomic diffusivity over several orders of magnitude using traditional methods. In those alloys it is not required to prevent crystallization by application of very high cooling rates. It is thus reasonable to assume that during such slow cooling processes (10^{-1}-10^{2} K/min), the system is able to continuously probe the equilibrium states of the supercooled liquid. As stressed in our main article, the case of AgInSbTe differs greatly from that, since an extremely high cooling rate is required to avoid crystallization during the quenching of the liquid phase (10^{9}-10^{10} K/s). Under these conditions it is instead reasonable that a glass is formed at a quite high temperature ($T_g \approx$ 550K).

Nevertheless, as an additional argument we show in figure 6S that the approach proposed by Ediger *et al.* cannot be valid in the case of melt-quenched AgInSbTe. We have modified equation (1) in the temperature range below 1.2 T_g assigning an exponent ξ to η that varies between 1 and 0.6. The fragility was fixed at $m = 130$ so that the experimental data at high temperatures are at least roughly met. The MYEGA model has been used to describe the temperature dependence of η. The discrepancy between our experimental data and the calculated values is clearly visible. Trying to

better reproduce an Arrhenius shape by reducing the value for the fragility m even deteriorates the fitting with the experimental data. This is because the whole curve would move down in the range of the investigated temperatures since the viscosity already increases more strongly at higher temperatures (that is what a smaller fragility would imply).

SUPPLEMENTARY NOTE 3: Temperature dependence of viscosity

Over the last decades several (three-parameter) equations have been proposed to fit the temperature dependence of the viscosity in the regime of the supercooled liquid[40]. In supplementary figure S3 we have plotted the temperature dependence of viscosity for the supercooled liquid of AgInSbTe according to the traditionally used Vogel-Fulcher-Tamman (VFT) model or, alternatively, employing the formula developed by Mauro and colleagues (MYEGA) to better describe especially the behavior of *fragile* liquids[38] (Because of the latter we believe the MYEGA model is more appropriate to cope with our data implying high fragilities, and thus only the MYEGA model is used for the fits in figure 4 of the main article):

$$\log_{10}(\eta(T)) = \log_{10}(\eta_\infty) + (12 - \log_{10}(\eta_\infty))\frac{T_g}{T}\exp\left[\left(\frac{m}{12 - \log_{10}(\eta_\infty)} - 1\right)\left(\frac{T_g}{T} - 1\right)\right] \qquad (S2)$$

The glass transition temperature T_g has been fixed to 443K, as measured by conventional DSC (at a heating rate of $\dot{T} = 40\mathrm{K/min}$) on an as-deposited and then relaxed AgInSbTe sample[39], whereas the parameter η_∞ has been chosen to fit the experimentally determined viscosity of the liquid phase $\eta_l = 2.09\ 10^{-3}\mathrm{Pa\ s}$ at $T = T_m = 808\mathrm{K}$ [26]. We have plotted two different curves based on the MYEGA formula changing the value of fragility $m = [\partial \log_{10}(\eta)/\partial(T/T_g)]_{T=T_g}$ from 100 to 130 (the corresponding values for η_∞ are -3.26 and -2.83, respectively). In the case of the VFT model the temperature dependence of viscosity can be rewritten as

$$\log_{10}(\eta(T)) = \log_{10}(\eta_\infty) + \frac{(12 - \log_{10}(\eta_\infty))^2}{m\frac{T_g}{T} + (12 - \log_{10}(\eta_\infty))} \qquad (S3)$$

with all parameters defined in the same way as previously when discussing Mauro's formula. The VFT equation is generally less successful to describe the behavior of fragile liquids [38, 40]. In fact, in our case, we had to assume a fragility of $m \cong 190$ (η_∞ ~-4.2) in order to let the curve in supplementary figure S3 touch our data on melt-quenched AgInSbTe. This value is larger than the reported theoretical maximum for the kinetic fragility ($m \cong 175$)[36].

SUPPLEMENTARY NOTE 4:

In the following we provide further information and more detailed discussions that serve to validate the correctness of our method used to determine the crystal growth velocity of melt-quenched amorphous AgInSbTe.

Determination of the temperature profile during the laser irradiation process

Meaningful data for crystal growth velocities can be obtained from the recovery time of the local reflectivity of a sample if the latter is significantly longer than the time it takes for the sample to cool down to the base temperature of the substrate, at which point the isothermal experiment is meant to take place. To check this condition we simulated the thermal response of our sample to laser heating by employing finite element method (see Methods section of the main article for details). Figure 1S shows the result of a laser irradiation process performed tuning the laser power to 83mW and keeping the substrate at room temperature. The left panel shows a section of the sample at t = 29ns, in which the area at $T>T_m$ is delimited by a black line. The spatial temperature profile reproduces the Gaussian shape of the pump laser. The right panel describes the temperature temporal evolution in the centre of the amorphous bit and it is evident that the substrate temperature is reached again after around 100ns after the pump laser is switched off. As we have stressed in the main paper, to obtain meaningful data, the crystallization time must be longer than the quenching time and this condition is fulfilled for T lower than ~550K. To obtain a higher cooling rate, it could be possible to reduce the thickness of the capping layer. However, this approach greatly reduces the stability of the layer stack that starts to evaporate approaching 550K.

The computer simulations were also used to estimate the radius of the amorphous bit. The calculation depicted in supplementary figure S4 predicts a radius of around 300nm. TEM ex-situ analysis of an according melt-quenched amorphous mark produced at room temperature verifies the accuracy of the simulation (see TEM micrograph in supplementary figure S5).

Calculation of bit radius from reflectivity measurement

The crystal growth velocity is obtained by measuring the time evolution of the amorphous bit radius. As we show below, time resolved reflectivity data are used for this purpose. A sudden decrease in reflectivity is observed after the creation of the amorphous mark. This drop can be described by the equation

$$\Delta R = \frac{P_{am,bit} - P_c}{P_c} \qquad (S4),$$

where P_c and $P_{am,bit}$ correspond to the measured power of reflected laser light before and after the creation of the amorphous bit. In polar coordinates these powers can be expressed as

$$P = \int_0^{\infty}\int_0^{2\pi} I_{in}(r,\varphi)R(r,\varphi)rd\varphi dr \qquad (S5)$$

In our experiments the incident intensity of the probe laser, I_{in}, has a Gaussian shape with a width ω_0 and in case of a homogeneous sample the reflectivity, R, is a constant, which is true for the fully crystalline initial state. Hence the previous equation can be rewritten as

$$P = 2\pi I_0 R \int_0^{\infty} \exp\left(-\frac{2r^2}{\omega_0^2}\right) rdr = \frac{1}{2}\pi I_0 \omega_0^2 R = c_0 R \qquad (S6)$$

And then, in a completely crystalline film

$$P_c = c_0 R_c \qquad (S7)$$

Likewise, the power reflected by an amorphous bit of radius r_{am}, embedded in its crystalline phase, can be written as

$$P_{am,bit} = \int_0^{r_{am}} 2\pi I_0 R_{am} \exp\left(-\frac{2r^2}{\omega_0^2}\right) rdr + \int_{r_{am}}^{\infty} 2\pi I_0 R_c \exp\left(-\frac{2r^2}{\omega_0^2}\right) rdr =$$

$$= 2\pi I_0 \left(\frac{\omega_0^2}{4} R_{am} - \frac{\omega_0^2}{4} R_{am} \exp\left(-\frac{2r_{am}^2}{\omega_0^2}\right) + \frac{\omega_0^2}{4} R_c \exp\left(-\frac{2r_{am}^2}{\omega_0^2}\right) - 0\right) =$$

$$= \underbrace{\frac{1}{2}\pi I_0 \omega_0^2}_{c_0} \left(R_{am} - (R_{am} - R_c)\exp\left(-\frac{2r_{am}^2}{\omega_0^2}\right)\right) \qquad (S8)$$

where R_c and R_{am} are the reflectivities of the crystalline and of the amorphous phase, respectively. Equations (S7) and (S8) can be used to rewrite equation (S4) in such a way that the change in reflectivity ΔR can be related to the radius of the amorphous bit r_{am}

$$\Delta R(r_{am}) = \frac{c_0\left(R_{am} - (R_{am} - R_c)\exp\left(-\frac{2r_{am}^2}{\omega_0^2}\right)\right) - c_0 R_c}{c_0 R_c} =$$

$$= \frac{R_{am} - R_c}{R_c}\left(1 - \exp\left(-\frac{2r_{am}^2}{\omega_0^2}\right)\right) \qquad (S9)$$

Reversing the previous equation we obtain the following formula

$$r_{am} = \omega_0\sqrt{\frac{1}{2}\ln\left(\frac{R_c - R_{am}}{R_{am,bit} - R_{am}}\right)} \qquad (S10)$$

that is used to obtain the radius of the amorphous mark at the beginning of a recrystallization experiment. To test this approach, we have calculated the radius of an amorphous mark obtained by a pump pulse of 29ns, tuning the power to 83mW and keeping the sample substrate at room temperature. The results derived from reflectivity measurements are nicely in agreement with both our FEM calculations and the TEM analyses. Increasing the substrate temperature, ex-situ TEM analysis cannot be performed (due to the immediate start of recrystallization reducing the size of the amorphous mark) but the maximum size of the amorphous bit can be estimated from the measured reflectivity drop using equation (S10) and the FEM simulation code. Supplementary Figure S6 shows the result of the simulation assuming a temperature of the substrate equal to 533K. A radius of about ~650nm is expected, in good agreement with the value obtained by the analysis of the reflectivity curve reported in the fig.1 of the main article.

SUPPLEMENTARY NOTE 5: TEM tilt series of isothermally recrystallized region

TEM bright field images of the investigated AgInSbTe samples show a very complex contrast variation, which is caused by a local variation of the Bragg contrast of individual regions in the crystalline grains. Two components of these contrast variations lead to the observed intensity distribution; (1) a mottled contrast which can be attributed to growth defects and inhomogeneity caused by the interface roughness between the AgInSbTe film and the capping layers and (2) bend contours which give the traces of the crystal regions in which the Bragg condition is fulfilled in a bent region of the TEM sample. Both effects will have a pronounced influence on the contrast observable in TEM images, which can easily be understood from the fact that the grains have a very high diameter/thickness ratio, with crystal sizes frequently exceeding one micrometer in diameter at a nominal thickness of only 30 nm. In polycrystalline samples, Bragg contrasts and bend contours can be used to determine crystal sizes and to obtain maps of the arrangement of individual grains. However, in the present case, the complex contrast cannot readily be interpreted in that way. Thus, in order to get a deeper and more quantitative insight in the crystal arrangement in a recrystallized area, a tilt series covering a tilt range of +/- 20 degree at an increment of 1 degree was acquired. The tilt series can be seen in supplementary movie 2. As expected, the bend contours move across individual grains, as is best seen for the larger grains. Furthermore, the visibility of individual grains varies strongly across the series, which gives the opportunity to identify individual grains with a much lower uncertainty than from an individual frame. In Supplementary Figure S7, we show the image obtained at zero tilt with an overlay of all the individual grains, which we have been able to identify with the help of the tilt series. The remaining unidentified areas are also crystalline, but the crystal size cannot be determined unequivocally. In the isothermally annealed region, the amorphized and (at 533K) recrystallized bit should be about in the centre of the frame, however there is no indication whatsoever that the bit shows a structural difference compared to its environment. As the bit size has about the same diameter as the average crystal diameter, an obvious interpretation of these findings is that the bit was recrystallized by a growth dominated

regime in which the neighbouring grains grow back into the amorphous region and virtually restore the original grain arrangement. On the other hand, obviously we cannot fully exclude the formation of a single nucleus, which by growth transfers the bit into a single new grain. However, since the *in situ*-experiments at even lower temperature show that recrystallization is clearly growth dominated, we assume that, in accordance with the thermodynamic principles, the recrystallization in the isothermal ex situ case also occurs via expansion of the neighbouring grains.
